# Good season ahead

**DOI:** 10.3325/cmj.2011.52.6

**Published:** 2011-02

**Authors:** Ana Marušić

Just as for people, a new year is a good time for a journal to stop, look back, and start a new beginning, hoping for a good season ahead. This is what we have been doing each year in the *Croatian Medical Journal* for the last twenty years. We will probably never stop doing this, even when we become an august journal, because the world of scientific publishing rapidly changes. This year, we present a new design of the journal’s website and remind our readers about new editorial policies on declaring authorship and conflict of interest.

After the testing period for the common conflict of interest reporting form of the International Committee of Medical Journal Editors (ICMJE) (1,2), we and other member journals of the ICMJE created an updated reporting form (3), which is now available at *http://www.icmje.org/updated_coi.pdf*. We have been asking our authors to fill out the form since 2009 but starting from this year we will publish their declaration in the journal. You will find it at the end of each article, together with other declarations and acknowledgments. We believe that conflict of interest, both of authors and editors, is an important issue (4,5), and will keep developing the optimal ways of declaring and managing it. During the testing period of the ICMJE reporting form for conflict of interest, we and other editors noticed that specialized terminology of the financial conflict of interest was not always clear, not only to those whose mother tongue is not English but also to many native English speakers. To clarify this terminology, we created a glossary, which is available at ICMJE Web site. The Croatian translation of the glossary is also available at *http://www.icmje.org/coi_glossary_croatian.pdf.* We recommend to our future authors to consult this document before they fill out the declaration form, so that the declaration published with their manuscript is accurate and clear.

From this issue on, we will also publish authors’ declaration on their authorship contributions. Since the first proposal for contributions disclosure in medical journals (6), we have been debating whether to publish the declarations we received because we knew that many authors did not satisfy the ICMJE authorship criteria according to their declared contribution. We extensively researched the authorship issue (7-11) and discovered that the declaration forms, used as self-reports on past behavior by authors as respondents, were not a reliable instrument to make conclusions on authorship. This is the reason why we decided against the use of common declaration forms that list possible contributions relevant to the ICMJE criteria. Instead, we ask each author a simple question: “Why do you think you deserve authorship of the submitted manuscript?”. You will be able to read our authors’ narratives from this issue on – sometimes brief and succinct and sometimes providing a more detailed explanation of the work done.

All details on new policies are available in our revised Guidelines for Authors, which are published in this issue (12) and are always available online at *http://www.cmj.hr/Guidelines/Guidelines.pdf.* We thank both the authors and the readers for their extra effort in supplying and assessing these new pieces of information – they will hopefully contribute to the quality and transparency of health research.
